# Rhesus Monkeys' Valuation of Vocalizations during a Free-Choice Task

**DOI:** 10.1371/journal.pone.0007834

**Published:** 2009-11-18

**Authors:** Brian E. Russ, Yale E. Cohen

**Affiliations:** 1 Department of Psychology, Harvard University, Cambridge, Massachusetts, United States of America; 2 Department of Otorhinolaryngology: Head and Neck Surgery, University of Pennsylvania School of Medicine, Philadelphia, Pennsylvania, United States of America; L'université Pierre et Marie Curie, France

## Abstract

Adaptive behavior requires that animals integrate current and past information with their decision-making. One important type of information is auditory-communication signals (i.e., species-specific vocalizations). Here, we tested how rhesus monkeys incorporate the opportunity to listen to different species-specific vocalizations into their decision-making processes. In particular, we tested how monkeys value these vocalizations relative to the opportunity to get a juice reward. To test this hypothesis, monkeys chose one of two targets to get a varying juice reward; at one of those targets, in addition to the juice reward, a vocalization was presented. By titrating the juice amounts at the two targets, we quantified the relationship between the monkeys' juice choices relative to the opportunity to listen to a vocalization. We found that, rhesus were not willing to give up a large juice reward to listen to vocalizations indicating that, relative to a juice reward, listening to vocalizations has a low value.

## Introduction

Adaptive behavior requires that animals form decisions that optimize potential rewards and minimize potential punishment [1], [2], [3]. To form these decisions, animals integrate their previous experience with the current context, along with other factors. One of the more important factors is the integration of social information [4], [5], [6]. For instance, rhesus monkeys when given a choice between receiving a large juice reward or viewing socially salient stimuli, choose to view the salient visual stimuli [4].

Successful adaptation to the environment also requires that humans and animals integrate information that is gathered from other sensory systems. One of the more important sources of information is auditory-communication signals (i.e., vocalizations) [7], [8], [9], [10], [11], [12]. Vocalizations are important for adaptive behavior since they transmit information about the identity and the age of the caller and often provide information about sex and emotional or motivational state [7], [13]. Some vocalizations transmit information about objects and events in the environment such as the type of predator, social relationships, or food quality [7], [13], [14].

Here, we tested how rhesus monkeys incorporate the opportunity to listen to different species-specific vocalizations into their decision-making processes. In particular, we tested whether rhesus would forgo a larger reward to hear a vocalization. This issue was addressed by having monkeys participate in a behavioral paradigm that quantified the preferences of rhesus to listen to vocalizations relative to the opportunity to obtain a juice reward; this paradigm is a modification of one used successfully to test monkeys preferences to view visual stimuli [4]. We found that, contrary to our predicted hypothesis and contrary to salient visual stimuli [4], rhesus were not willing to give up a large juice reward to listen to vocalizations indicating that, relative to a juice reward, listening to vocalizations has a low value.

## Results

**Figure 1:**
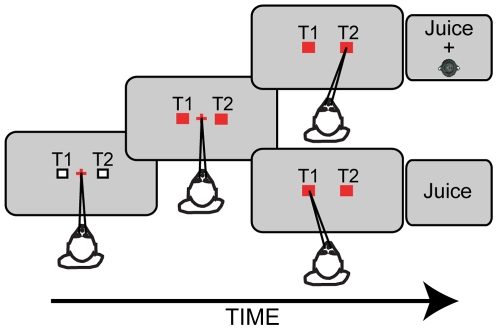
Schematic of the pay-per-listen task. After fixating a central LED, two peripheral visual targets (T1 and T2) were illuminated. Following a delay, the central LED was extinguished, and the monkey shifted their gaze to either target to receive a juice reward. On non-baseline trials, when the monkey chose T2, an auditory stimulus was also presented; the schematic illustrates these non-baseline trials.

Monkeys participated in the “pay-per-listen” task [4]. In this task, monkeys chose one of two targets (T1 or T2) to get a juice reward (Fig. 1). Following a period of “baseline”-data collection, a species-specific vocalization was introduced into the task; this auditory stimulus could be one of five exemplars from one of six different classes of vocalizations. Specifically, if the monkeys chose target T2, they were also presented with a species-specific vocalization; the vocalization was presented at T2, the location that the monkey was attending. By titrating the T1∶T2 juice ratio, we quantified the relationship between the monkeys' juice choices relative to the opportunity to listen to a vocalization. See **Materials and Methods** for more details.

**Figure 2:**
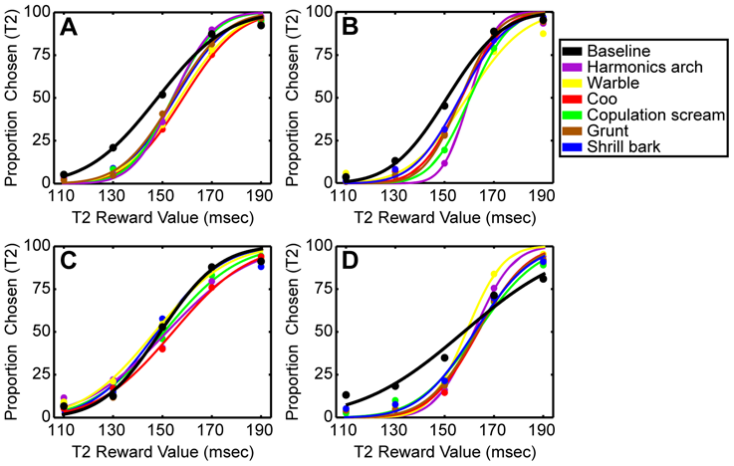
The average behavioral choices for (A) monkey E, (B) monkey H. (C) monkey Z, and (D) monkey B. Their choices are plotted as a function of the proportion of T2 choices for each T2 reward value. These behavioral data were fit with a cumulative Gaussian function and the resulting curves are plotted along with the behavioral choices. The colored data points and lines represent the monkeys' choices for each vocalization block.

Figure 2 shows the monkeys' behavioral performance during the pay-per-listen task. The monkeys reliably discriminated between the different reward ratios during the baseline blocks (black data). More specifically, the monkeys rarely chose T2 when more juice was offered at T1 than at T2 (i.e., those trials when the T1∶T2 reward ratio was 170∶130 msec or 190∶110 msec). In contrast, the monkeys often chose T2 when more juice was offered at T2 than at T1 (i.e., 130∶170 msec or 110∶190 msec trials). T2 was chosen at intermediate rate when equal amounts of juice were offered at T1 and T2 (i.e., when the T1∶T2 reward ratio was 150∶150 msec).

To quantify the effect that the vocalization had on the monkeys' choice of T2, we fit each block of baseline data with a cumulative Gaussian function and quantified from this curve each monkey's point of subjective equality (PSE) [15]. The PSE was the point where the monkey considered choice of target T1 and target T2 to be equivalent. For 3 of the 4 monkeys, the PSEs were not reliably different than 150 msec (i.e., the time duration that gave equivalent amounts of juice at both targets; see Table 1). For the fourth monkey, monkey B, there was a slight bias to choose target T1 (Table 1).

**Table 1:** Distribution of baseline, vocalization, and grand PSE values as a function of each tested monkey.

Monkey	Baseline	Harmonic arch	Warble	Coo	Copulation scream	Grunt	Shrill bark	Grand Vocalization
E	148.03	154.30 *	157.13*	*158.41	154.12*	154.56*	155.25*	155.52*
H	151.04	160.06*	159.09*	156.89	160.32*	156.63	156.32	158.23*
Z	149.19	151.81	146.95	154.28	150.61	149.34	147.87	149.91
B	157.78*	161.82	158.80	163.60	163.19	163.23	162.20	162.03*

* indicates PSE values that differ from reliably from chance (p<0.05).

**Figure 3 pone-0007834-g003:**
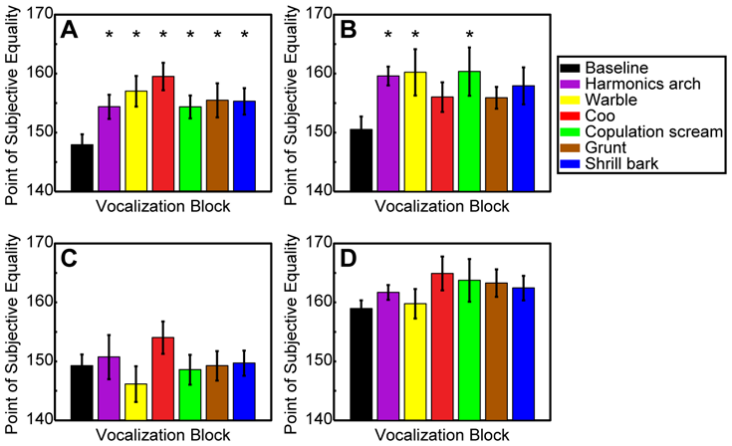
Baseline and vocalization PSEs for (A) monkey E, (B) monkey H, (C) monkey Z, and (D) monkey B. The black bar is the baseline PSE, whereas each colored bar represents the each individual vocalization PSE value. * represent vocalization PSEs that are significantly different (p<0.05) from the baseline PSE (black bars). Error bars represent one standard error.

How were the PSE values altered during the auditory blocks when sets of vocalizations were presented at the target T2? The behavioral curves from each vocalization block are shown in Figure 2 and the PSE values from each vocalization block are shown in Table 1. In general, the monkeys' behavioral performance was quite variable. For monkey E, we found that the baseline PSE was reliably less (p<0.05) than each of the vocalization mean PSEs (Table 1; and Fig. 3A); a lower PSE indicates that, on average, the T2 reward value had to be larger, relative to baseline, for the monkey to chose this target. For monkeys H and B, we found that only a subset of the vocalization PSEs were reliably different (specifically larger) than the baseline PSE (Table 1 and Fig. 3B and C). Finally, for monkey Z, we could not identify a reliable difference between any of the vocalization PSEs and the baseline PSE (Table 1; and Fig. 3D).

**Figure 4:**
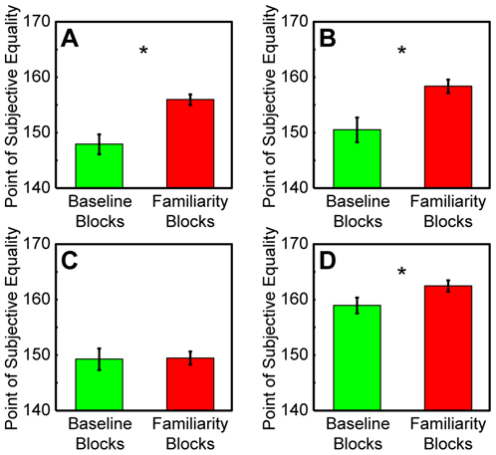
Baseline and the grand vocalization PSE for (A) monkey E, (B) monkey H, (C) monkey Z, and (D) monkey B. The baseline PSEs are shown in green and the grand PSEs are shown in red. * represent vocalization PSEs which are significantly different from the baseline PSE (p<0.05). Error bars represent one standard error.

To further test the effect that the vocalizations had on the monkeys' T2 choices, we calculated a “grand” PSE that combined the data from all of the vocalization blocks (see **Materials and Methods**). The results of this analysis are shown in Figure 4. Three of the four monkeys had grand-vocalization PSEs that were reliably larger (p<0.05) than their baseline PSEs. A binomial test indicated that the probability of finding this proportion of grand-vocalization PSES that were greater than the baseline vocalizations was significantly greater than chance. As noted, an increase in PSE value indicated that we needed to offer the monkeys a larger reward for them to choose T2 (and listen to a vocalization).

## Discussion

During baseline trials, when presented with two targets (T1 and T2) that delivered different volumes of juice, monkeys reliably chose the target that delivered the larger volume of juice (black data in Fig. 2). The pairing of a vocalization with the monkeys' choice of target T2 altered the monkeys' preferences: on average, larger quantities of juice had to be offered at target T2 for the monkeys to choose this target (Figs. 2–4). Whereas there was considerable variance in this effect—both across monkeys and across vocalization class (Fig. 3), a more consistent pattern (i.e., requiring more juice to choose target T2) was found when the vocalizations were treated as a single group (Fig. 4).

How do our results compare with those by Deaner et al. who paired a visual stimulus with target T2 [4]? With a visual stimulus, monkeys altered their target preferences (and hence, the volume of juice that was delivered) depending on the social value of the stimulus. Specifically, the monkeys forfeited juice when given the opportunity to view pictures that had high-social value (e.g., high-status male monkeys) at target T2. However, when pictures with low-social value (e.g., low-status monkeys) were paired with target T2, the monkeys had to be offered larger quantities of juice to choose target T2; a neutral grey square did not alter the preference for target T2.

Given this pattern of findings from Deaner et al., we hypothesize that, within the context of the task, the value of listening to vocalizations has a low value. This finding may be analogous to Deaner et al's finding that when target T2 was paired with pictures of low-status monkeys, larger quantities juice had to be offered for the monkeys to choose target T2 [4].

Why would, in the context of the task, vocalizations have a low value? In other words, why would monkeys not choose to orient their gaze to a location to hear a vocalization? Several non-exclusive possibilities emerge. First, whereas vocalizations are communication signals, the intention of a vocalizing monkey may not always be to transmit information to a listener [13], [14]. Second, auditory-communication signals are not always veridical [16]. Third, solitary individuals that are not part of a group dynamic can elicit vocalizations [7], [13], [14]. Together, these possibilities suggest that, within the context of the task, it may not be adaptive for a thirsty monkey that is trying to maximize its intake of juice to attend to signals that may not be transmitting information to them or to those that provide non-veridical information.

However, this low-value hypothesis does not imply that vocalizations are *not* a salient stimulus for rhesus, even for those that are laboratory housed [17]. Indeed, vocalizations play a fundamental role in the socioecology of several species of non-human primates [7], [13], [18] since vocalizations convey information about the identity and the age of the caller, emotional/affective state, and information about objects and events in the environment (e.g., kinds of predators, social relationships, or food quality). We are simply postulating that within this task and the goals of the monkey to get juice, the opportunity to hear vocalizations may not have a high value. In fact, previous research has suggested that rhesus preferentially pull a lever to hear auditory stimuli as opposed to receiving no reward at all [19], [20]. The current study, on the other hand, suggests that rhesus are more interested in maximizing their juice rewards as compared to hearing species-specific vocalizations.

Could the value of the vocalizations be increased within the context of the pay-for-listen task? One strategy might be to present vocalizations from known con-specifics of different ranks, analogous to Deaner et al. [4]. A second strategy might be to present the vocalizations in a more ecologically-valid manner or to systematically manipulate the validity. However, regardless of these manipulations or others, it may very well be that vocalizations will always be relatively low value to monkeys engaged in “foraging” for juice rewards.

### Potential Confounds

Three differences between our study and Deaner et al. [4] might be confounds in our study. First, relative to Deaner et al., we had less experimental variability. In our study, we had 5 vocalization-exemplars/pool and changed the T1∶T2 reward ratios every 50 trials. In contrast, in Deaner et al., they had 20 visual-exemplars/pool and changed the reward ratios every 30 trials. This decreased variability in our study might have habituated the monkeys to the repeated presentations of the vocalizations [17]. Consequently, their interest in the vocalization presented at T2 might have waned over time. To test for this possibility, we compared the monkeys' behavioral performance from the first half of data collection to their performance during the second half (Fig. S1). These analyses indicated that the monkeys' PSEs were not reliably different between the first and second halves of data collection. Consequently, the amount of experimental variability cannot wholly account for differences between the data sets.

Another issue is that the social relationships of the monkeys and/or the relevance of the social-communication signals were different in our study than in Deaner et al. For example, the identities of the individuals eliciting the vocalizations were unknown to our monkeys, whereas in contrast, in Deaner et al., they were known. Since rhesus monkeys in laboratory-based colonies do not produce the entire repertoire of vocalizations, we could not obtain exemplars elicited by familiar individuals from all 6 classes of vocalizations that were used in this study. However, this issue may not be limiting since we have shown that rhesus in laboratory-based colonies and the wild respond to and categorize many classes of vocalizations in a similar fashion [21]. A second related issue is that our monkeys lived in a colony that minimized social interactions, whereas the Deaner et al. monkeys lived in a rich social environment. Consequently, it is plausible that our monkeys' social environment might have altered the monkeys' responses to the vocalizations within the context of the pay-per-listen task.

Nevertheless, as a test for familiarity, we repeated the pay-for-listen task using familiar auditory stimuli. Since we were unable to create an appropriate database of vocalizations from known individuals, we substituted exemplars of spoken words into the pay-for-listen task from a known human (i.e., the monkey's caretaker) and an unknown human. Whereas this strategy has obvious weaknesses, we felt this approach was reasonable (1) since monkeys appear to have the capacity to discriminate between different caretaker's voices [22] and (2) since neural activity in rhesus is not necessarily modulated preferentially by familiar vocalizations [23]. We found that the monkeys' preferences to choose target T2 was not reliably different (p>0.05) when the auditory stimulus was spoken words from a familiar speaker than when it was spoken words from an unfamiliar speaker (Fig. S2). This result suggests that familiarity cannot wholly account for differences between Deaner al.'s findings and ours as familiarity had did not have a reliable effect on our monkeys' preferences.

A final issue to consider is whether the interaction between the visual and auditory stimuli might have affected the monkey's capacity to process the vocalizations. Since the monkeys were trained to attend to the T1 or T2 targets to receive a reward, it is possible that their processing of (or interest in) the vocalizations was compromised in favor of the visual targets [24], [25], [26], [27], [28]. However, this compromised processing cannot fully explain our findings. If the monkeys were unable to process the vocalizations, then we would predict that the vocalizations would not alter the monkeys' choices of target T2. However, this prediction was not observed since we did see reliable changes in the monkeys' choices (Fig. 4).

## Materials and Methods

Four rhesus macaques (*Macaca mulatta*) participated in this experiment, two males and two females. Under isofluorane anesthesia, the monkeys were implanted with a scleral search coil and a head-positioning cylinder. All of the rhesus monkeys were housed in individual home cages in a colony room with up to eight other rhesus. For the majority of the study period, the two female rhesus were pair-housed. All of the rhesus were on water restriction while participating in the experiment and received all of their fluid due to the participation in this experiment; a minimum daily level of fluid intake for adequate hydration was determined separately for each animal. The Dartmouth Institutional Animal Care and Use Committee approved all of the experimental protocols.

### Experimental Rig

Behavioral sessions were conducted in a darkened room with sound-attenuating walls. The walls were covered with anechoic foam insulation (Sonomatt, Auralex). When inside the room, the monkeys were seated in the primate chair and placed in front of a stimulus array; since the room was darkened, the speakers producing the auditory stimuli were not visible to the monkeys. The primate chair was placed in the center of a 1-m diameter, two-dimensional, magnetic coil (Riverbend Instruments) that was part of the eye-position monitoring system [29]. Eye position was sampled with an analog-to-digital converter (PXI-6052E, National Instruments Inc.) at a rate of 1.0 kHz. The monkeys were monitored during all sessions with an infrared camera.

The stimulus array consisted of three red LEDs that formed a line centered on the monkey. The two LEDs were each centered on a speaker (PLX32, Pyle). The LEDs were 1.2 m above the floor, which was the approximate eye level of the monkeys. Relative to the monkey's position in the room, the LED-to-LED separation was 10° in azimuth.

### Auditory Stimuli

**Figure 5:**
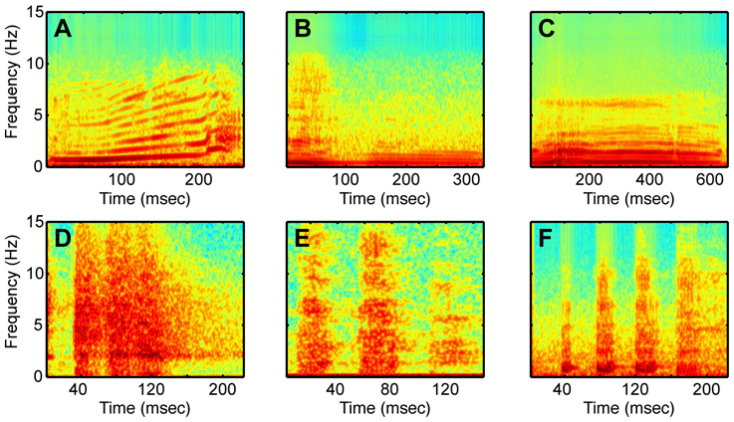
Representative exemplars from each vocalization class. Each panel shows the spectrogram of a (**A**) harmonic arch, (**B**) warble, (**C**) coo, (**D**) copulation scream, (**E**) grunt, and (**F**) shrill bark.

The auditory stimuli were rhesus-macaque species-specific vocalizations (SSVs). In this study, we had five exemplars from six different classes of vocalizations: “copulation screams”, “warbles”, “grunts”, “barks”, “coos”, and “harmonic arches”. Each vocalization class had different putative referential meanings or different affective levels [30], [31]. The vocalizations were recorded as part of an earlier set of studies [30]. A spectrogram of one exemplar from each vocalization pool is shown in Figure 5. A “pool” of stimuli consisted of the five exemplars from the same stimulus class (e.g., 5 different coos). The vocalization exemplars within each class were generated from different callers to maximize their variability and to help maintain the monkeys' interest.

The average auditory stimulus was 426 msec in length with a range of 105 msec (one of the shrill-bark exemplars) to 1396 msec (one of the copulation-scream exemplars). Each auditory stimulus was presented at a sound level of 65 dB SPL. All of the stimuli were recorded to disk and sampled at 50 kHz. The stimuli were presented through a D/A converter (DA1, Tucker Davis Technologies), an anti-aliasing filter (FT6-92, Tucker Davis Technologies), and an amplifier (SA1, Tucker Davis Technologies, and MPA-250, Radio Shack).

### Behavioral Task: Pay-Per-Listen Task

The “pay-per-listen” task was adapted from Deaner et al. [4] and is schematized in Figure 1. The task began with the monkey fixating the central LED. Following a 500-msec delay, the two peripheral LEDs (target “T1” and target “T2”) were illuminated; operationally, T1 was the LED to the monkey's left, relative to the monkey's position, whereas T2 was the LED to the monkey's right. After another additional 300 msec, the central LED was turned off, and the monkey shifted its gaze to either T1 or T2 to be rewarded. If the monkey chose T1 and maintained their gaze at it for 500 msec, it received a juice reward. If T2 was chosen, an auditory stimulus was presented from this location while the monkey received their juice reward. Each auditory stimulus was present only once per trial. Importantly, during the initial training and during some portions of data collection (see below), an auditory stimulus was not presented when the monkey chose T2.

The amount of the juice reward varied randomly on a block-by-block basis. “Reward blocks” were pseudo-randomly chosen and consisted of 50 trials in which the juice-reward ratio remained constant for targets T1 and T2. Within a reward block, the T1∶T2 ratios were (in msec) 110∶190, 130∶170, 150∶150, 170∶130, or 190∶110. If the solenoid opened for 110 msec, the monkey received ∼0.07 ml of juice, whereas when the solenoid opened for 190 msec the monkey received ∼0.12 ml of juice reward.

An “auditory block” consisted of five reward blocks; that is, 250 trials. Within an auditory block, one of five exemplars from the same auditory pool was randomly chosen on a trial-by-trial basis. The auditory pool was selected randomly prior to the start of each auditory block.

### Training on the Pay-Per-Listen Task

The monkeys were first trained to differentiate between the largest T1∶T2 reward pairing (i.e., 110∶190 msec or 190∶110 msec). Once the monkeys reliably learned to choose the target (T1 or T2) with the larger reward amount, we then introduced the reward pairings that had a smaller T1∶T2 reward pairing (130∶170 msec or 170∶130 msec). The monkeys trained on blocks of these four sets of reward pairings until the monkey could reliably chose the larger reward value during randomly presented trial blocks. Finally, we introduced the equal reward pairing (150∶150 msec).

### Behavioral-Testing Strategy

Following training, we collected baseline blocks of behavioral data. During these baseline blocks, we did not present an auditory stimulus when the monkeys chose target T2. After 15–20 baseline blocks, an auditory stimulus was presented when the monkeys chose T2 (i.e., auditory blocks were introduced).

### Data Analysis

Each monkey's performance was quantified by plotting the relationship between the T2 reward value and the proportion of times that the monkey chose T2. These data were then fit with a cumulative Gaussian function using the Curve Fitting Toolbox in the Matlab programming environment (Mathworks Inc). Data were plotted and fit as a function of each auditory block. From these curves, we calculated the monkeys' point of subjective equality [15] (PSE), which was the point where the monkey considered choosing the T1 and T2 to be the same. Operationally, the PSE was defined as the point on the fitted curve when the proportion of times that the monkey chose T2 was 50%. The PSE was calculated independently for each baseline block and for each auditory block. If the PSE was greater than the maximum reward value (190 msec) or less than the minimum reward value (110 msec), this block was excluded from further analyses, because these PSE values fell out of the range of possible choices: less than 5% of blocks had such extreme PSE values.

Distributions of PSE values were calculated as a function of the baseline blocks, each vocalization pool. Also, a “grand” distribution vocalization PSE was calculated from the individual distributions of vocalization PSE values.

A t-test tested whether the distribution of baseline PSEs was reliably different, at a level of p<0.05, than the distribution of vocalization PSEs or the grand distribution PSE.

## Supporting Information

Figure S1 Grand vocalization PSE for (A) monkey E, (B) monkey H, (C) monkey Z, and (D) monkey B as a function of the first half and second half of data collection. Error bars represent one standard error.
(0.17 MB TIF)

Figure S2 Familiar and unfamiliar human spoken word PSEs for (A) monkey E, (B) monkey H, (C) monkey Z, and (D) monkey B. * represent vocalization PSEs which are significantly different from the baseline PSE (p<0.05). Error bars represent one standard error.
(0.17 MB TIF)
