# Supplementary figures and images for: Rhesus Monkeys' Valuation of Vocalizations during a Free-Choice Task
Source: PLoS One. 2009 Nov 18;4(11):e7834. doi: 10.1371/journal.pone.0007834 (PMC2771902; doi:10.1371/journal.pone.0007834)

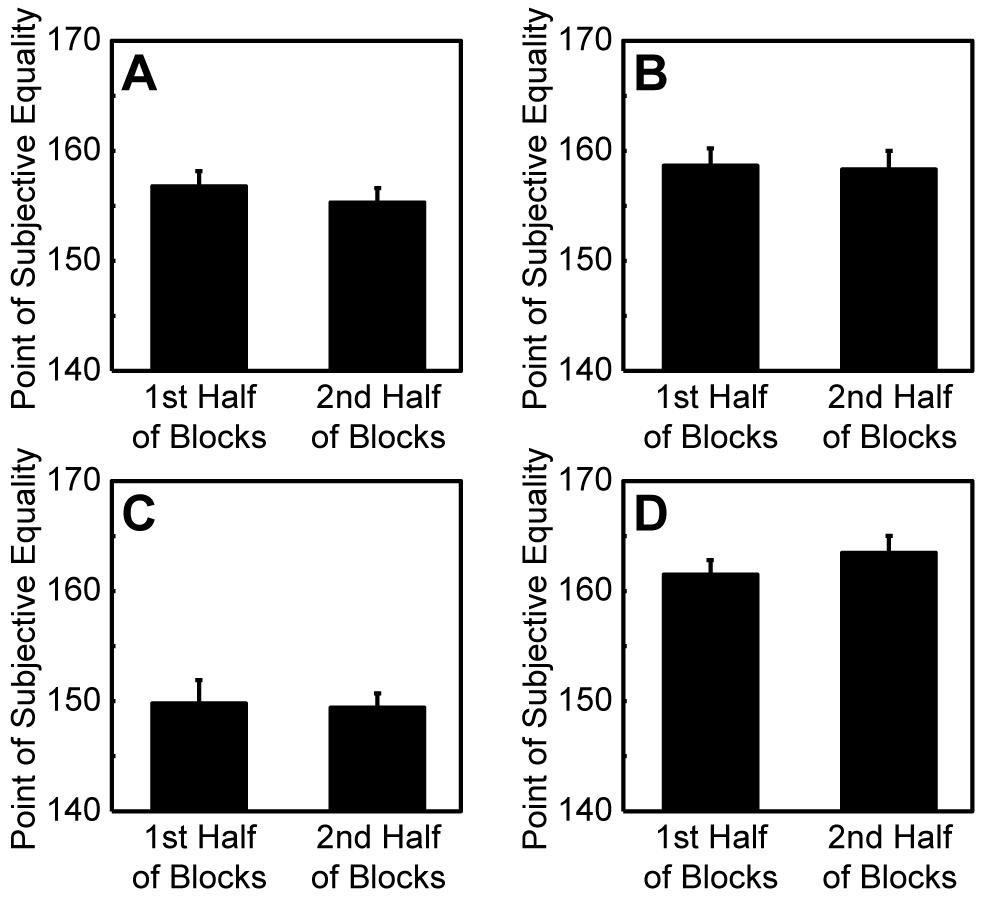

Supplement: Figure S1 — Grand vocalization PSE for (A) monkey E, (B) monkey H, (C) monkey Z, and (D) monkey B as a function of the first half and second half of data collection. Error bars represent one standard error. (0.17 MB TIF) [file pone.0007834.s001.tif]

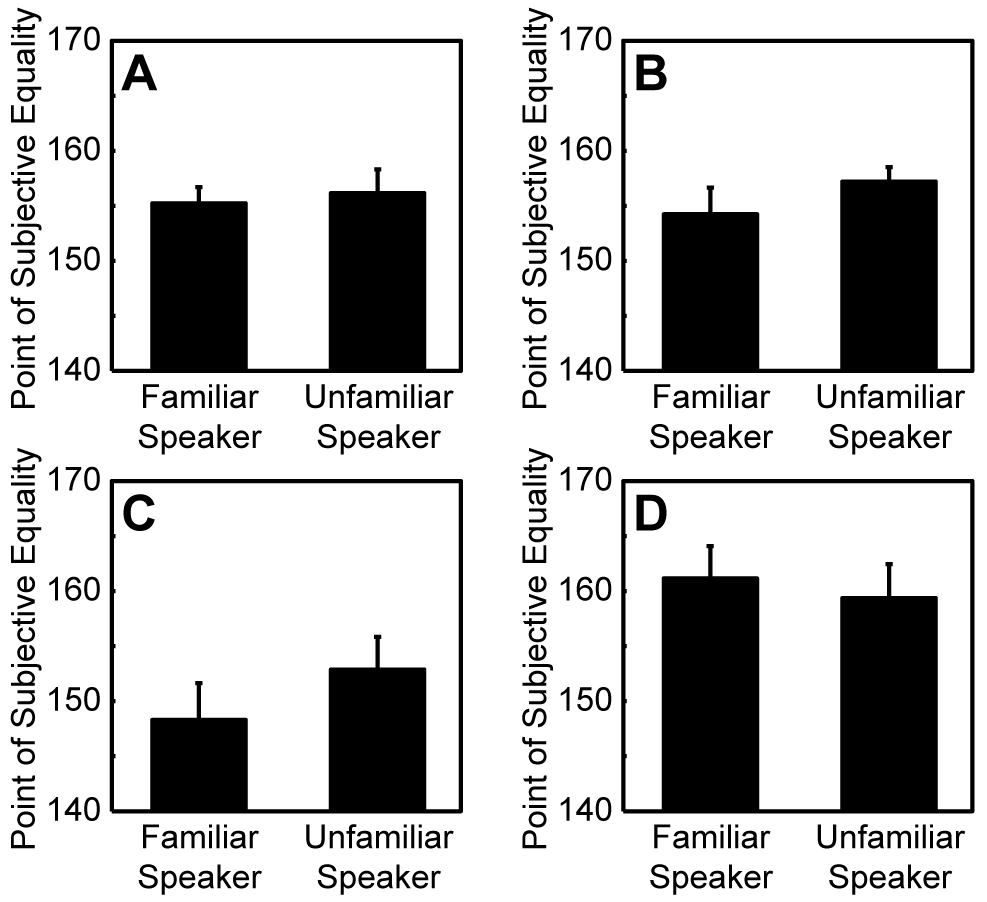

Supplement: Figure S2 — Familiar and unfamiliar human spoken word PSEs for (A) monkey E, (B) monkey H, (C) monkey Z, and (D) monkey B. * represent vocalization PSEs which are significantly different from the baseline PSE (p<0.05). Error bars represent one standard error. (0.17 MB TIF) [file pone.0007834.s002.tif]
